# Substance Use in the Club Scene of Rome: A Pilot Study

**DOI:** 10.1155/2014/617546

**Published:** 2014-08-28

**Authors:** Alessandro Emiliano Vento, Giovanni Martinotti, Eduardo Cinosi, Matteo Lupi, Tiziano Acciavatti, Dario Carrus, Rita Santacroce, Eleonora Chillemi, Ludovica Bonifaci, Massimo di Giannantonio, Ornella Corazza, Fabrizio Schifano

**Affiliations:** ^1^Asl RMC, Osservatorio sulle Dipendenze e sui Disturbi Psichici Sotto Soglia, 00100 Rome, Italy; ^2^Department of Neuroscience and Imaging, University “G.d'Annunzio”, 66100 Chieti, Italy; ^3^Asl VT, Osservatorio sulle Dipendenze e sui Disturbi Psichici Sotto Soglia, 00100 Rome, Italy; ^4^Libera Università Maria SS. Assunta, 00100 Rome, Italy; ^5^School of Life and Medical Sciences, University of Hertfordshire, Herts SG13, UK

## Abstract

*Objective*. Over the last few years, a wide number of unregulated substances have been marketed on the Web and in smart and head shops; they are usually advertised as legal alternatives to commonly known drugs and are defined as “smart drugs,” “legal highs,” and “novel psychoactive substances” (NPS). Aim of our work is to describe use habits and distribution of NPS in a population of young adults in Rome club scene.* Methods*. A self-administered questionnaire was proposed to subjects over 18 years of age at the entrance of 5 nightclubs in Rome. Socioeconomic characteristics and substance use were investigated.* Results*. Preliminary results give evidence that 78% of respondents have a lifetime history of NPS use. In addition, 56% of the sample has consumed illicit drugs in the past and 39% has used psychoactive substances in the 12 hours preceding the questionnaire administration.* Conclusions*. A significant proportion of subjects report use of novel psychoactive substances; traditional illicit drugs consumption, particularly cocaine, appears to be very high as well in the club scene. These data highlight a serious public health challenge, since pharmacological, toxicological, and psychopathological effects linked to interactions among all these substances may be unpredictable and sometimes fatal in vulnerable individuals.

## 1. Introduction

So-called “club drugs,” psychoactive substances associated with club and rave cultures, proliferated in the 1990s and remained key substances for years among youth and young adults [1]. The U.S. National Institute on Drug Abuse (NIDA) in its “Community Alert on Club Drugs” identified ecstasy (3,4-methylenedioxymethamphetamine or MDMA), Gamma-hydroxybutyrate (GHB), ketamine, Rohypnol (flunitrazepam), methamphetamine, and lysergic acid diethylamide (LSD) as “club drugs” [2], while the U.S. Office of National Drug Control Policy described only four specific club drugs: MDMA, GHB, ketamine, and Rohypnol [3].

Epidemiological studies have shown that factors associated with recreational nightlife activities, such as music preference and choice of the venue, may be considered as relevant predictors of illegal drug use in several European countries [4]. Similarly, in the US, Moore and Miles have found an association between substance use and alternative music styles in the electronic music scene [5].

It is becoming increasingly clear that the drugs used in clubs are heterogeneous and ever-changing [6]; moreover, during the last few years, a wide number of unregulated natural and synthetic New Psychoactive Substances (NPS) have made their appearance on the market [7].

NPS are drugs of abuse, either in a pure form or in a preparation, that are not controlled by the 1961 Single Convention on Narcotic Drugs or the 1971 Convention on Psychotropic Substances, but that may pose a serious public health threat. In this context, the term “new” does not necessarily refer to newly synthesized molecules, but mainly to substances that have recently become available [7]. Many of these substances were, in fact, firstly synthesized and patented in the 1970s or even earlier, but only recently their chemistry or process of synthesis has been slightly modified to produce effects similar to known illicit substances. On the basis of the above-mentioned definition, the European Monitoring Centre for Drugs and Drug Addiction (EMCDDA) and the European Police Office identified the following groups of substances, covered by the Early Warning System on NPS [8]:

- *phenethylamines*, which encompass a wide range of substances that may exhibit stimulant, entactogenic, or hallucinogenic effects;
- *tryptamines*, which include a number of substances that have predominantly hallucinogenic effects;
- * piperazines*, represented, inter alia, by m-chlorophenylpiperazine (mCPP) and BZP, both of which are central nervous system stimulants;
- * synthetic cathinones*, which have stimulant effects; the main cathinone derivatives are the semisynthetic methcathinone and the synthetic compounds mephedrone, methylone, and methylenedioxypyrovalerone (MDPV);
- *synthetic cannabinoids*, which are functionally similar to Δ9-tetrahydrocannabinol (THC), the active compound of cannabis.

Other substances reported by the Early Warning System include various plant-derived and synthetic psychoactive substances (e.g., indanes, benzodifuranyls, narcotic analgesics, synthetic cocaine derivatives, ketamine and phencyclidine derivatives), which do not strictly belong to any of the previously mentioned drug groups. A number of medicinal products and derivatives are also included [9].

NPS are rapidly appearing in more and more sophisticated forms and often remain unregulated for a long period [7]. EMCDDA stated that, in 2012, 280 potentially harmful “legal highs” have been produced in Europe alone [8]. These substances are often synthesized in underground laboratories, simply modifying the molecular structure of controlled drugs, hence raising concerns related to the potential presence of contaminating agents [10]. The significant informational, promotional, and distributional capacity of the Internet plays an important role in the NPS market: global web-based marketing and distribution, distinct from illegal street markets, have developed in the past years [9]. The number of online shops providing customers with NPS in European Union countries has increased from 170 in January 2010 to 314 in January 2011, and 693 in January 2012 [8]. The Internet offers many advantages to NPS suppliers, as it allows access to a vast number of potential users, and suppliers do not need large upfront investments and can retain some level of anonymity. In addition, suppliers may be able to bypass the laws of different countries, thus making enforcement or legal action in response to their activities very difficult. It is also important to point out that, in many cases, sellers fail to list ingredients, side effects, and drug interactions of the advertised products [7]. Moreover, the Internet serves as a repository of information for several groups of people. Drug users can obtain information through online forums, chat rooms, and blogs and find out about new products. They can also share with other users their experiences and the effects of the substances, as well as the recommended sources and ways of delivery [10]. The possibility of purchasing NPS from websites makes these drugs easily available to vulnerable individuals, including children and adolescents. These subjects may also be encouraged by diffuse online comments/messages/videos related to NPS intake experiences. This may be an issue of concern, if one considers that an estimated 61% of young Europeans aged between 15 and 24 years typically quote the Internet as a potential source of information on illicit drugs [11]. Moreover, the current legal status of a number of NPS may arguably facilitate increasing levels of popularity of these drugs and may affect as well the users' perception of risks associated with their consumption. The idea that legality can equate with safety still remains well grounded amongst some recreational users [12].

The limited information available suggests that NPS spread at a global level is far from negligible, and, excluding cannabis from the analysis, it comes close to, or even exceeds, the spread of several controlled drugs. NPS have been reported in many countries in recent years. What is actually known today, however, maybe just the very tip of the iceberg, as systematic studies on the diffusion of NPS do not exist [9], except for single substances [13, 14].

The consumption of NPS in Italy in 2011 was estimated to be about 1%, placing the country 27th out of 28 European countries in the ranking of NPS use [14]. In the last years, the Italian Department of Drug Control has promoted the development of a concise update on the main features of identified NPS, developing as well a series of strategic guidelines, objectives, and actions in order to begin constructing a response to tackle this emerging issue [15]. Although the Italian data are encouraging and show the effectiveness of the countermeasures taken to deal with this new phenomenon (e.g., to contrast NPS sale in smart shops and sex shops), the presence of toxic products still new on the market remains an open issue to which great attention is paid. Moreover, while estimates on general population are essential to better illustrate drug trends, it is also important to identify and assess consumption prevalence among key groups and target populations.

Aim of our study was to describe use habits and distribution of NPS and other substances in a population of young adults. Considering that surveys undertaken in nightclubs and other nighttime economy venues are a good source of data in order to assess the use of recreational drugs in a high-prevalence use population [16], we selected a sample of young adults involved in Rome nightclub scene, investigating substance use as well as possible psychotropic and side effects.

## 2. Methods

### 2.1. Study Participants

A questionnaire on recreational substances misuse has been proposed between September and November 2013 to a population sample (18–30 years old) in 5 nightclubs in Rome. Potential study participants were approached by a member of the research team at the entrance or in the smoking/chill-out area inside the clubs. The self-administered questionnaire was collected in anonymous way by our team of psychologists and psychiatrists, with the support of a peer group working.

In the first part, the questionnaire included socioeconomic characteristics (age, gender, job status, lifestyle) and perceived mental stress. Written informed consent was systematically obtained for every subject, according to the Declaration of Helsinki.

### 2.2. Previous Illicit Drugs Use

Study participants were asked whether they had used, in the past, any illicit drug among heroin (diacetylmorphine) and other opiates (opium, morphine, codeine, heroin, fentanyl, methadone, meperidine, L-alpha-acetylmethadol [LAAM]), cannabis (marijuana, hashish), cocaine and derivatives (e.g., crack), amphetamines, methamphetamine, hallucinogens (lysergic acid diethylamide [LSD], mescaline, psilocybin), and stimulatory hallucinogens (MDMA [ecstasy], phencyclidine [PCP]). Common street names of illicit drugs, both single and in combination (e.g., speed, speedball, spaceball), were also listed. For illicit drugs with multiple routes of administration (ROA), interviewees were asked to specify the ROA and pharmaceutical form (e.g., powder, pill, dust).

Moreover, study participants were asked if they had ever abused alcohol (according to the DSM IV-TR criteria) with the above-mentioned illicit drugs and, in case of positive response, they were asked to specify which drugs and the frequency of this behavior.

### 2.3. Previous Use of Novel Psychoactive Substances and/or Clubs Drugs

Study participants were asked whether they had used a NPS and/or a club drug in the past. Listed substances included synthetic cannabinoids, Gamma-hydroxybutyrate (GHB), mephedrone, ketamine,* Salvia divinorum*, amyl nitrite, and psilocybin. Common street or on-line names of NPS (e.g., poppers, liquid ecstasy, liquid X, magic mushrooms) were included among the questionnaire options as well. If the participants answered positively, they were asked to specify if the chosen substance was a powder, a pill, or another type of NPS.

### 2.4. Current Use of Psychoactive Substances and Alcohol

Study participants were asked to indicate whether they had used psychoactive substances in the 12 hours preceding the questionnaire administration. If the answer was positive, they were asked to specify if they had assumed also alcohol as well.

### 2.5. Data Analysis

Data are reported as means, standard deviation, and percentages where appropriate. Statistical comparison of the proportions of those reporting use on the night of the survey was undertaken by Chi-square (*χ* ^2^). SPSS version 14.0 was used for all analyses.

## 3. Results

### 3.1. Participant Characteristics

This sample was composed of 273 subjects. Regarding the sociodemographic characteristics, mean age of participants was 25.4 years, 53% were males and 47% females, and all were residents in the municipality of Rome. 62% of subjects lived with parents, 13% alone and the remaining 25% with other people. With regard to employment status, 27% declared to have a job, 46% were students, 10% were working students, and, finally, 17% were unemployed.

### 3.2. Previous Illicit Drugs Use

The prevalence of lifetime previous illicit drugs use was 56% (*n*. 153), and 48% of the subjects claimed to have consumed alcohol together with illegal psychoactive substances at least once.

### 3.3. Previous Use of Novel Psychoactive Substances and/or Clubs Drugs

Surprisingly, 78% of respondents declared a lifetime previous use of NPS/club drugs, such as amyl nitrite (45%), synthetic cannabinoids (35%), lysergic acid diethylamide (LSD) (24%), mephedrone (18.8%), ketamine (18%), Gamma-hydroxybutyrate (GHB) (10.2%), psilocybin (4%), and* Salvia divinorum* (3.2%) (see Table 1).

### 3.4. Current Use of Psychoactive Substances and Alcohol

**Figure 1:**
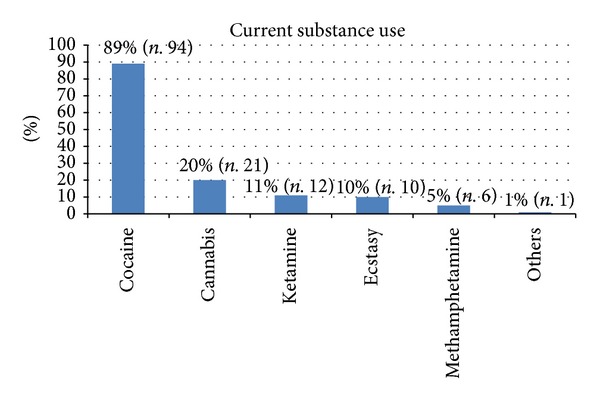
Percentages of substance use within the group of current users.

At the time of the questionnaire administration, 39% of the sample (*n*. 106) claimed to have assumed psychoactive substances in the previous 12 hours. Among the most prevalent substances, the percentages were distributed as listed: cocaine 89% (*n*. 94), cannabis 20% (*n*. 21), ketamine 11% (*n*. 12), ecstasy 10% (*n*. 11), methamphetamine 5% (*n*. 6). Other psychoactive substances had a prevalence of use of less than 1% (*n*. 1) (see Figure 1). Overall, on the night of the survey, cocaine had a prevalence higher than cannabis (*P* < 0.001), ketamine (*P* < 0.001), ecstasy (*P* < 0.001), or methamphetamine (*P* < 0.001).

Moreover, the sample showed high polydrug use frequencies: among psychoactive substances consumers, the totality (100%) claimed a concurrent alcohol consumption, and 31% (12% of the total sample) (*n*. 33) claimed the assumption of two or more illicit drugs.

## 4. Discussion

In the surveyed sample, a significant fraction (78%) reported a previous lifetime history of use of one or more NPS (“legal high”) and/or club drugs. The lifetime use of amyl nitrite was particularly high, probably for its easy availability and its sexual enhancing properties [17]. However, the use of newer psychoactive substances was also very high (see Table 1 for details). There are two main possible ways to explain this finding: (1) a recent spread of knowledge and consumption of NPS in the Italian youth population or (2) despite an overall limited use, a widespread consumption in peculiar subpopulations. However, notwithstanding this impressive datum, there was no evidence of recent use (on the night of the survey) for new psychoactive substances. On the contrary, a very high consumption was highlighted for cocaine, followed by cannabis and other substances commonly rubricated as “club drugs” (ketamine, ecstasy, methamphetamine) [2, 3]. These results contrast with other studies on the recreational drug scene in the UK, which reported a fairly significant consumption of mephedrone [18–20], Gamma-butyrolactone (GBL) [21], and amyl nitrite [20]. Our results are more similar to reports on the nightlife scenes of US [22] and continental Europe [23]. Cocaine is easily available and in recent years has become cheaper than in the past [24]: these reasons can help to explain its high prevalence of use.

Our results evidence high rates of polydrug use in the subpopulation of nightclubs attendees. This could determine a serious public health challenge, since pharmacological, toxicological, and psychopathological effects due to interactions between these substances may be unpredictable and fatal as well in vulnerable individuals. As previously stated, very few pharmacological/toxicological data are available in the peer-reviewed scientific literature, with the limited knowledge being mostly restricted to preclinical studies. Moreover, a polydrug use may lead to deep neurobiochemical central nervous system alterations, which may make these individuals extremely difficult to be pharmacologically treated, even by expert mental health professionals.

Club drugs have also an important social impact in terms of crimes and violence [25], and they may contribute to the global burden of diseases, together with mental disorders and traditional illicit drugs (e.g., heroin) [26]. In 2010, illicit drug dependence accounted for 20 million DALYs (disability-adjusted life years), being responsible for 0,8% (0.6–1.0) of global all-cause DALYs [27]. At present, however, the overall dependence liability of these substances seems lower than that of heroin and amphetamines [27].

## 5. Limitations

This is a pilot study with a small sample size: for definitive conclusions it is necessary to expand the sample. However, our sample size is comparable with that considered in other in situ nighttime economy surveys [28]. Increasing the available data would allow having greater statistical significance, also in order to identify drugs complications and interactions, to verify the indirect effects of substances on the social and working capacity and, maybe, to evaluate the consequences of their long-term assumption. An intrinsic limitation is that drugs abuse in club scene appears to be jeopardized, and it is difficult to extend the results obtained at a venue to other venues or countries. Another limitation is that subjects may not be aware of what drug(s) they had actually used; however every effort was made in the attempt to be accurate (e.g., use of substances street names, specification of formulation and route of administration).

## 6. Conclusions

This survey has shown that although a significant proportion of individuals report a lifetime use of NPS, these drugs have little significance in the recreational drugs scene. Therefore, to investigate the recreational drugs scene might not be the better strategy to monitor the extent of NPS consumption in the European Union and elsewhere. However, multicenter large-scale studies need to be carried out. Again, it is here suggested that better international collaboration levels are needed to tackle the new and fast growing phenomenon of NPS availability from the web and other channels of supply.

Regarding traditional illicit drugs, cocaine misuse is very high in club culture, significantly higher than substances usually identified as club drugs (MDMA, ketamine, methamphetamine). Moreover, health and other professionals should be accurately informed about the trend of misuse of multiple psychoactive substances in association with alcohol.

Further studies are needed to design better intervention strategies in subjects with problematic recreational drugs use within the nightclub environment.

## Figures and Tables

**Table 1:** 

Novel psychoactive substance/club drugs	Lifetime use (% values of the whole sample)	Absolute values
Synthetic cannabinoid	35	96
Gamma-hydroxybutyrate (GHB)	10.2	28
Mephedrone	18.8	51
Ketamine	18	49
*Salvia divinorum *	3.2	9
Amyl nitrite (poppers)	45	123
Lysergic acid diethylamide (LSD)	24	66
Psilocybin	4	11
